# Optogenetic Dissection of Neural Circuits Underlying Stress-Induced Mood Disorders

**DOI:** 10.3389/fpsyg.2021.600999

**Published:** 2021-06-17

**Authors:** Qing Liu, Zhinuo Zhang, Wenjuan Zhang

**Affiliations:** ^1^College of Education and Technology, Zhejiang University of Technology, Hangzhou, China; ^2^Mental Health Education Center, Xidian University, Xi'an, China

**Keywords:** mood disorders, optogenetics, neural circuit activity, stress, projection

## Abstract

**Objectives:** This review aims to (i) summarize the literature on optogenetic applications of different stress-induced mood disorder models of the medial prefrontal cortex (mPFC) and its projection circuits, and (ii) examine methodological variability across the literature and how such variations may influence the underlying circuits of stress-induced mood disorders.

**Methods:** A variety of databases (PubMed, Web of Science, Elsevier, Springer, and Wiley) were systematically searched to identify optogenetic studies that applied to mood disorders in the context of stress.

**Results:** Eleven studies on optogenetic stimulation of the mPFC and the effect of its efferent circuitry on anxiety- and depression-like behaviors in different rodent models were selected. The results showed that the optogenetics (i) can provide insights into the underlying circuits of mood disorders in the context of stress (ii) and also points out new therapeutic strategies for treating mood disorders.

**Conclusions:** These findings indicate a clear role for the mPFC in social avoidance, and highlight the central role of stress reactivity circuitry that may be targeted for the treatment of stress-induced mood disorders.

## Introduction

Prolonged exposure to severe social stress is a critical risk factor for psychiatric disorders such as major depressive disorders (Bosch-Bouju et al., 2016). Depression is highly comorbid with anxiety; mood and anxiety disorders can disrupt the basic functions of individuals' lives and are among the leading causes of disability (Russo and Nestler, 2013; Vos et al., 2015). The complexity and diversity of anxiety and depression symptoms, as well as the heterogeneity of the brain regions involved, are major obstacle to understanding the neural circuits that mediate anxiety and mood disorders (Sapolsky, 2016). However, optogenetics, a technique developed in the early 2000 s, has proved effective in the study and treatment of anxiety and depression-like behaviors in animal models (Boyden et al., 2005; Hare and Duman, 2020). Several methodological breakthroughs have enhanced our understanding of the neural mechanisms underlying stress and related disorders, such as genetic methods for altered gene expression in discrete brain regions, or cell populations (Covington et al., 2010; Son et al., 2018). More recently, optogenetics methods have enabled the stimulation or inhibition of activity among a target cell population in animal exposed to stress (Carlson et al., 2017; Kataoka et al., 2020). Since the discovery and implementation of optogenetics, several investigators have used this technique in combination with behavioral tests for depression-like and anxiety-like behaviors in order to delineate the neural pathways underlying stress-related disorders (Walsh and Han, 2014; Fakhoury, 2020).

In humans, social stress induces several health problems, including anxiety and depression. Numerous studies have identified the medial prefontal cortex (mPFC) as dysfunctional in stress-related disorders, mPFC dysfunctionalities include alterations in structure, in markers of glutamatergic and gamma-aminobutyric acid (GABA) neurotransmission, and in connectivity with downstream structures (Duman et al., 2019). Animal models provide further evidence of chronic stress exposure on changes in affective-like behaviors. Among these, the social defeat stress paradigm (SDS) is well-characterized and has been investigated in various research fields, particularly in studies concerning mood disorders (Berton, 2006; Krishnan et al., 2007; Golden et al., 2011; Toyoda, 2017). SDS is a resident-intruder paradigm involving physical fights among animals (e.g., rodents), followed by single housing of the defeated intruder rats using a sensory contact model, where animals are housed in a semi-protected compartment of the cage of the resident (Pryce and Fuchs, 2017). Once daily, a partition separating the two compartments is briefly removed, allowing a physical conflict and defeat exposure. After replacement of the separator, experimental animals experience a continuous threat of defeat by visual and olfactory cues (Patel et al., 2019). The advantage of this model is that defeated rodents exhibit signs of depression-like behavior, including anhedonia, increased anxiety, and decreased locomotor activity, thus reproducing the symptoms of depression observed in human patients (Hultman et al., 2016). One prominent disadvantage of the SDS model is that most of the test subjects have historically been male rodents, although recent advances have provided for a wider range of test subjects to be studied, including females (Newman et al., 2019). In these cases, stressors mostly involve noxious or physically distressing stimuli such as tail suspension, forced swimming, physical restraint or immobilization, or subjection to tests, for example, the sucrose preference test (SPT) or the open field test (Cheng et al., 2019; Cui et al., 2020).

The forced swim test (FST) is performed by placing a rodent (rat or mouse) in a container filled with water to a sufficient depth that the animals cannot support themselves and must choose between active swimming and climbing, or inactive floating (Slattery and Cryan, 2012). One optogenetic study directly stimulated the descending projection neurons in the mPFC in mice engineered to express Channelrhodopsin-2 (Chr2) in layer V pyramidal neurons (Thy1-Chr2 mice). This was done to model antidepressant-like behavior in mice subjected to PFC stimulation (Kumar et al., 2013). The tail suspension test (TST) paradigm involves the exposure of mice to the inescapable stress of being suspended from the tail for 6 min with the behavioral outcome measure of immobility time (Iñiguez et al., 2018). The elevated plus maze (EPM) paradigm consists of a plus-shaped maze on an elevated platform. The maze contains two open arms without walls and two closed arms, which are enclosed by high walls; rodents are found to instinctively avoid the open arms. In the open field test (OPT), which consists of an enclosure with high walls, rodents spend more time exploring the walled periphery of the open field and avoiding its exposed center (Richardson-Jones et al., 2011).

In each of these tests, rodent inactivity has been described as reflecting anhedonia and social avoidance (depression-like behaviors), while reduced exploration suggests enhanced anxiety levels (Tye and Deisseroth, 2012; Adhikari, 2014). However, more recent interpretations have focused on the transition between adaptive and maladaptive coping that was impacted by prior experiences; and rodent non-social models, such as FST, have become paradigms to investigate the mechanistic underpinning of stress coping and adaptation (de Kloet and Molendijk, 2016). Stimulation of glutamatergic and GABAergic neurons in the mPFC of mice exposed to SDS led to reduced social avoidance, as well as anhedonia as determined in the SPT, which are consistent with an antidepressant response (Covington et al., 2010). Therefore, with the ongoing development of optogenetic tools to probe the *in vivo* functions of ever-more specific circuits, a summary of the psychosocial stress rodent models and non-social stressor models with optogenetic stimulation on neural mechanism investigation of stress-related disorders is required. The purpose of the present study is thus to (i) summarize the literature on optogenetic applications of different rodent stress models of the mPFC and its projection circuits, and (ii) examine methodological variability across the literature and how such variations may influence the underlying circuits of stress-induced mood disorders.

## Methods

### Retrieval Strategy and Data Extraction

A systematic search of the online databases—PubMed, Web of Science, Elsevier, Springer, and Willey was conducted to retrieve articles published until February 15, 2020. The search strategy used involved the use of subject words and free words, using the following words without date restrictions: (“optogenetic” OR “optogenetics” OR “optogenetic techniques” OR “optogenetic technique” OR “technique, optogenetic” OR “techniques, optogenetic”) AND (“mood disorder” OR “disorder, mood” OR “disorders, mood” OR “mood disorder” OR “affective disorders” OR “affective disorder” OR “disorder, affective” OR “disorders, affective”) AND (“prefrontal cortex”) AND (“stress”). We also looked closely at the reference lists of published literature for more potential research works. We excluded literature reviews, meta-analyses, case studies, conference abstracts, practical guides, and book articles. Second, each study identified from the targeted databases was purposefully screened according to the title, abstract, and keywords of the literature, combined with the inclusion and exclusion criteria.

**Figure 1:**
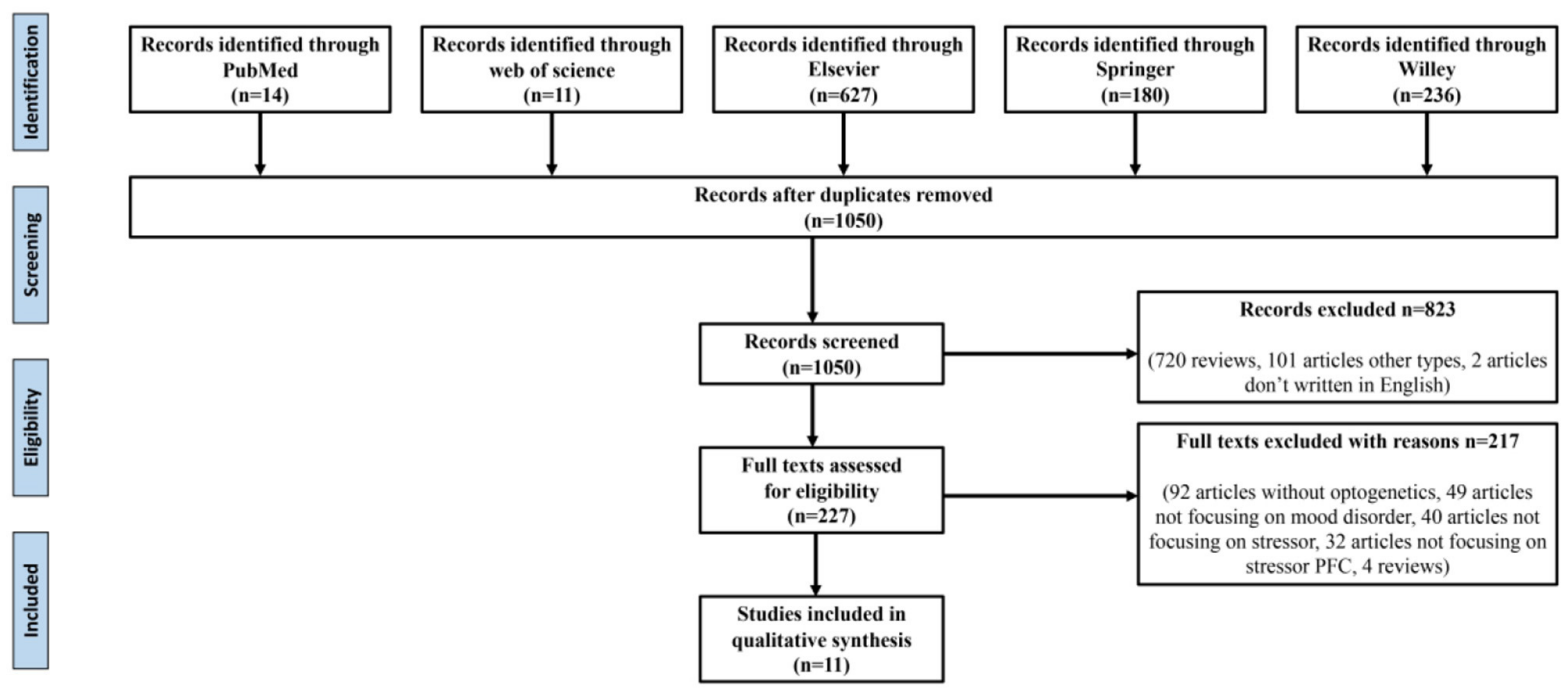
Data retrieval process.

The reference retrieval strategy is shown in Figure 1. The following data were extracted: (1) the study's title, first author, publication year, and (2) research characteristics, namely stress-induced types involving brain regions and specific neuron types, optogenetic stimulation types, behavior test types, their results, and preliminary findings.

## Results And Discussions

As shown in Figure 1, after eliminating duplicates, 1,050 records were generated from the five databases after the initial search. After a preliminary screening of titles, keywords, abstracts, and types, 823 studies were excluded. We carefully investigated their contents of the remaining 227 studies. Finally, 11 of these met all the inclusion criteria and our research conditions (Table 1).

**Table 1:** The underlying circuits of stress-induced mood disorders and its influencing factors.

	**Stress paradigms**	**Region targeted**	**Neuron**	**Opsin(s)**	**Manipulation (Activation/Inhibition)**	**Test/behaviors**	**Findings**	**References**
VTA to mPFC	CSDS (10days)	VTA	DA	ChR2	OA (20 Hz, 5 spikes, 10 s interval)	SPT↓ SI↓	Increase depression-like phenotypes with activation	Chaudhury et al., 2013
	CSDS (10days)	VTA to mPFC	DA	ChR2	OA (20 Hz,8 s, 2 s interval)	SPT↔ SI↔	No depressive effect with activation	Chaudhury et al., 2013
	CSDS (10days)	VTA to mPFC	DA	NpHR	OI (20 Hz, 8 s, 2 s interval)	SI↑ SPT↔	Increased depression-like phenotypes with inhibition	Chaudhury et al., 2013
mPFC	SDS	mPFC	N/A	ChR2	OA (100 Hz, 1 s, 3 s interval)	SPT↑ SI↑	Reduced depression-like phenotypes with activation	Covington et al., 2010
	SDS	mPFC	N/A	ChR2	OA (100 Hz, 1 s, 3 s interval)	EPM↔ OPT↔	No anxiogenic effect with activation	Covington et al., 2010
	SDS	mPFC	N/A	ChR2	OA (100 Hz, 1 s, 3 s interval)	Social recognition test↔	No cognitive effect with activation	Covington et al., 2010
	FST	mPFC	5-HT	ChR2	OA (20 Hz 5 ms)	FST↔ OFT↔	No depressive effect with activation	Warden et al., 2012
	susceptible mice	mPFC (PrL)	N/A	ChR2	OA (2.5 mW, 1 min)	OFT↑	Have anti-depressive effect with activation	Kumar et al., 2013
	susceptible mice	mPFC (PrL)	N/A	ChR2	OA (2.0 mW, 5 min)	FST↑ OFT↑	Have anti-depressive effect with activation	Kumar et al., 2013
	CSDS (15 days)	mPFC (PrL)	N/A	ChR2	OA (2.0 mW, 5 min)	EPM↑	Reduced anxiolytic-like phenotypes with activation	Kumar et al., 2013
	CSDS (15 days)	mPFC (PrL)	N/A	ChR2	OA (2.0 mW, 5 min)	SI↔ OFT↔	No depressive effect with activation	Kumar et al., 2013
	susceptible mice	mPFC (PrL)	N/A	ChR2	OA (100 Hz 40 ms, 3 s interval)	N/A	Have anti-depressive effect with activation	Vialou et al., 2014
	TST, electric shock	mPFC (PL)	N/A	C1V1	OA (2 mW/pulses, 46 pulses/30 ms, 3s, 36 ms interval)	Decision test↓	Reduced social decision-making behaviors with activation	Friedman et al., 2017
	TST, electric shock	mPFC(PL)	N/A	eArch	OI (2 mW/pulses, 1 pulse 3 s)	Decision test↑	Increased social decision-making behaviors with activation	Friedman et al., 2017
	TST	mPFC	Glu	ChR2	OA high-frequency (100 Hz, 1 s, 3 s interval)	TST↑	Reduced depression-like phenotypes with activation	Son et al., 2018
	FST	mPFC	Drd1 DA	ChR2	OA (5 mW/side, 10 Hz, 15 ms, 1 min on/1 min off, 60 min)	FST↑ SPT↔	Reduced depression-like phenotypes with activation	Hare et al., 2019
	FST	mPFC	Drd1 DA	ChR2	OA (5 mW/side, 10 Hz, 15 ms, 1 min on/1 min off, 60 min)	EPM↑ NSF↔	No anxiogenic effect with activation	Hare et al., 2019
	FST	mPFC	Drd2 DA	ChR2	OA (5 mW/side, 10 Hz, 15 ms, 1 min on/1 min off, 60 min)	FST↔ SPT↔	No depressive effect with activation	Hare et al., 2019
	FST	mPFC	Drd2 DA	ChR2	OA (5 mW/side, 10 Hz, 15 ms, 1 min on/1 min off, 60 min)	EPM↓ NSF↔	Reduced anxiolytic-like phenotypes with activation	Hare et al., 2019
mPFC-AMY	CSDS (10 days)	mPFC (PrL)-BLA	Glu	ChR2	OA (100 Hz, 40 ms, 3 s interval)	SI↔ SPT↔	No depressive effect with activation	Vialou et al., 2014
	CSDS (10 days)	mPFC (PrL)-BLA	Glu	ChR2	OA (100 Hz, 40 ms, 3 s interval)	EPM↑	Reduced anxiolytic-like phenotypes with activation	Vialou et al., 2014
mPFC-Striatum	CSDS (10 days)	mPFC (PrL)-NAc	Glu	ChR2	OA (100 Hz, 40 ms, 3 s interval)	SI↑ SPT↑	Reduced depression-like phenotypes with activation	Vialou et al., 2014
	CSDS (10 days)	mPFC (PrL)-NAc	Glu	ChR2	OA (100 Hz, 40 ms, 3 s interval)	EPM↔	No anxiogenic effect with activation	Vialou et al., 2014
mPFC- Thal	FST	mPFC-LHB	N/A	ChR2	OA (20 Hz, 5 ms)	FST↓	Increased depression-like phenotypes with activation	Warden et al., 2012
	TST	mPFC (IL)-Thal	N/A	ChETA	OA (5 Hz, 15 ms interval/ 14.05 Hz)	TST↑ OFT↔	Reduced depression-like phenotypes with activation	Carlson et al., 2017
	SDS	DP/DTT → DMH	N/A	iChloC	OI (10 Hz, 30/180 s)	SI↑, OFT↑	Reduced depression-like phenotypes with inhibition	Kataoka et al., 2020
	susceptible mice	DP/DTT → DMH	Glu	ChIEF	OA (10 Hz, 30/180 s)	BAT↑	Induced psychosocial stress responses with inhibition	Kataoka et al., 2020
mPFC-DRN	FST	mPFC-DRN	Glu, GABA, 5-HT	ChR2	OA (20 Hz, 5 ms)	FST↑ OFT↑	Reduced depression-like phenotypes with activation	Warden et al., 2012
	CSDS (11days)	vmPFC-DRN	GABA	ChR2	OA (7.33 mW mm^−2^, 25 hz, 10 ms, 20 min)	SI↓	Increased depression-like phenotypes with activation	Challis et al., 2014
	CSDS (11days)	vmPFC-DRN	GABA	Arch	OI (7.05 mW mm^−2^, 20 min)	SI↑	Reduced depression-like phenotypes with inhibition	Challis et al., 2014

*AMY, amygdala; 5-HT, serotonergic nucleus; BAT, brown adipose tissue; BLA, basolateral amygdala; ChETA, channelrhodopsin-2 variant; ChR2, channelrhodopsin-2; CSDS, chronic social defeat stress; DA, dopamine; DP, dorsal peduncular cortex; DTT, dorsal tenia tecta; DMH, dorsomedial hypothalamus; EPM, Elevated plus maze; FST, forced swim test (non-social stress model); GABA, γ-aminobutyric acid; Glu, glutamatergic; IL, infralimbic cortex; LH, lateral habenula; NAc, nucleus accumbens; NSF, novelty suppressed feeding test; OPT, open field test; OA, optogenetic activation; OI, optogenetic inhibition; SDS, social defeat stress; SI, Social interaction test; SPT, sucrose preference test; THal, Thalamus; TST, tail suspension test (non-social stress model)*.

This systematic review seeks to summarize optogenetic applications to manipulate the mPFC and its circuitry in studies of stress-induced mood disorders. The systematic analysis showed that optogenetic stimulation of ventral tegmental area (VTA) projections to the mPFC decreased social avoidance in mice following an SDS paradigm, while the stimulation of mPFC projections to the BLA blocked social stress-induced behavioral deficits. These findings indicate a clear role for the mPFC in social avoidance and highlight a stress reactivity circuitry that may be targeted for the treatment of stress-induced mood disorders.

### Optogenetic Stimulation on VTA-mPFC Circuit: Afferent Circuitry of mPFC

As a central hub that receives input from cortical regions and sends outputs to a structure that regulates emotion, fear, and stress responses, the mPFC plays a critical role in behavior (Hare and Duman, 2020). The dopaminergic afferent to the mPFC appears to play a role in social avoidance. Induction of phasic firing in the VTA dopamine neurons of mice undergoing SDS paradigm rapidly induced a susceptible phenotype as measured by social avoidance and decreased sucrose preference (Chaudhury et al., 2013). Their results also showed that optogenetic inhibition of VTA neurons projecting to the mPFC led to increased susceptibility to stress, while optogenetic stimulation of VTA neurons projecting to the mPFC induced stress resilience. These findings revealed a novel firing pattern and neural circuit-specific mechanisms of depression.

### Optogenetic Stimulation of the mPFC Influences Anxiety-and Depression-Like Behaviors: Efferent Circuitry of mPFC

The manipulation of mPFC cell populations on anxiety and depression-like behaviors revealed the involvement of the mPFC in coping and social interactions. Depression-like behaviors incorporate a stressful challenge to assess active and inactive behavioral periods (Kumar et al., 2013; Son et al., 2018). Son and his colleagues (2018) investigated whether glutamate (Glu) and glutamine (Gln) levels and glutamatergic neuronal activity are altered in the mPFC of a chronic immobilization stress (CIS) induced animal model, and whether increments of glutamatergic activity in the mPFC could change depressive-like behaviors. They found that low Glu and Gln levels and low glutamatergic neuronal activity in the mPFC due to CIS induced hypoative Glnsythetase (GS) in depressed mice. Immobility was also reduced in the FST model. Kumar et al. (2013) showed that direct optogenetic stimulation of descending projection neurons in the PFC in mice engineered to express Chr2 in layer V pyramidal neurons (Thy1-Chr2 mice) models an antidepressant-like effect in mice subjected to FST. They also found that prefrontal cortex stimulation induces a long-lasting suppression of anxiety-like behavior in socially stressed Thy1-Chr2 mice, which means that the direct activation of cortical projection systems is sufficient to modulate activity across networks underlying affective regulation.

Covington et al. (2010) examined the immediate early genes (IEGs)zif268 (egr1), c-fos, and arc in the ventral portion of the mPFC of mice after chronic social defeat stress (CSDS, a mouse model of depression) and optogenetically drove “burst” patterns of mPFC cortical firing *in vivo* to observe the behavioral consequences. Their results showed that mice subjected to CSDS exhibited reduced levels of IEG expression in the mPFC, which indicates deficits in neuronal activity within this brain region. These results were in accordance with the significant reductions in IEG expression in prefrontal cortical tissue derived from clinically depressed humans. What is interesting in the study by Convington et al. was that the reduction of IEG neurons in the mPFC was not observed in defeated mice that escape the deleterious consequences of stress (resilient animals), indicating that the activity of the mPFC was a key determinant of depression-like behavior that only occurred in mice expressing strong depressive-like phenotype (susceptible animals).

### Optogenetic Stimulation of the mPFC-BLA Circuit

The projection from the mPFC to the BLA was stimulated using optogenetic tools in susceptible mice that were injected with cholecystokinin (CCK, transcription factor of FosB) in the mPFC (Vialou et al., 2014). The optogenetic stimulation of mPFC-BLA projections reversed CCK-induced social avoidance and sucrose preference and produced no anxiety-like behaviors. Moreover, it has been reported that dopamine receptor Drd1-expressing cells in the mPFC, which projected to the BLA, produced rapid and sustained antidepressant effects 7 days after photostimulation (Hare et al., 2019). These findings help in understanding the cellular target neurons in the mPFC and the downstream circuitry involved in rapid antidepressant responses.

### Optogenetic Stimulation on Other Circuits of mPFC

By leveraging optogenetic projecting-targeting to control cells with specific efferent wiring patterns, Warden et al. (2012) selectively activated mPFC cells projecting to the dorsal raphe nucleus (DRN) and observed that this induced a profound, rapid, and reversible effect on rats' decision to act in a challenging situation. The stimulation of mPFC-DRN projections induced antidepressant-like reduction in immobility during the FST, while the stimulation of mPFC terminals in the lateral habenula (LHB) increased immobility. Increases in immobility were also observed in mice subjected repeatedly using the TST and optogenetically stimulated cellular activity in mFPC and medial dorsal thalamus (MDT), which reflects a compensatory mechanism whereby the brain drives neural systems to counterbalance the effects of stress. Moreover, the mPFC projections to DRN have also been shown to bidirectionally modify social defeat outcomes. Challis et al. (2014) used an SDS model to induce social aversion in mice and then optogenetically stimulated or inhibited the mPFC-DRN pathway during sensory exposure to aggressors' cues and observed that increasing activity in this pathway enhanced subsequent social avoidance, while inhibition induced the opposite antidepressant-like effects. These results clarify the functional organization of mPFC-DRN pathways and indicate that top-down mPFC influences affect-regulating neuron output.

Immobility and social avoidance in mice were observed in one study using SDS and optogenetic inhibition of the mPFC to the dorsomedial hypothalamus (DMH) monosynaptic pathway (Kataoka et al., 2020). This mPFC-DMH circuitry-driven sympathetic and behavioral response using the SDS model indicated that neurons in the mPFC transmit psychological stress-driven glutamatergic signals to the DMH to elicit a variety of stress responses. Later studies have also observed aberrant decision-making under chronic stress by optogenetically stimulating the mPFC to the thalamus (thal) circuit in rats performing a cost-benefit test (Friedman et al., 2017). The high-cost/high-reward options were sharply increased in rats after a CSDS test, which suggests that the mPFC-thal circuit was critical for the induction of aberrant cost-benefit evaluation caused by chronic stress. Taken together, optogenetic techniques with high-fidelity control of neuronal activity in pre-clinical models have elucidated the contribution of the mPFC and its effect on anxiety and depression-like behaviors, and demonstrated the utility of optogenetic tools for determining likely sites of adaptation to stress experiences.

In this review, we summarized the rodent models and neural circuits of mood disorders studied using optogenetics in the context of stress. We found that optogentic stimulation of VTA projections to the mPFC decreased social avoidance in mice following an SDS paradigm, while the stimulation of mPFC projections to the BLA blocked social stress-induced behavioral deficits. These findings indicate a clear role for the mPFC in social avoidance and highlight a stress reactivity circuitry that may be targeted for the treatment of stress-induced mood disorders.

## Author Contributions

QL and ZZ selected the topic. All authors analyzed the data, wrote and revised the manuscript, and approved to the submitted version.

## Conflict of Interest

The authors declare that the research was conducted in the absence of any commercial or financial relationships that could be construed as a potential conflict of interest.

## References

[B1] Adhikari A. (2014). Distributed circuits underlying anxiety. Front. Behav. Neurosci. 8:112. 10.3389/fnbeh.2014.00112 24744710 PMC3978252

[B2] Berton O. (2006). Essential role of BDNF in the mesolimbic dopamine pathway in social defeat stress. Science 311, 864–868. 10.1126/science.1120972 16469931

[B3] Bosch-Bouju C. Larrieu T. Linders L. Manzoni O. J. Layé S. (2016). Endocannabinoid-mediated plasticity in nucleus accumbens controls vulnerability to anxiety after social defeat stress. Cell Rep. 16, 1237–1242. 10.1016/j.celrep.2016.06.082 27452462

[B4] Boyden E. S. Zhang F. Bamberg E. Nagel G. Deisseroth K. (2005). Millisecond-timescale, genetically targeted optical control of neural activity. Nat. Neurosci. 8, 1263–1268. 10.1038/nn1525 16116447

[B5] Carlson D. David L. K. Gallagher N. M. Vu M.-A. T. Shirley M. Hultman R.. (2017). Dynamically timed stimulation of corticolimbic circuitry activates a stress-compensatory pathway. Biol. Psychiatry. 82, 904–913. 10.1016/j.biopsych.2017.06.008 28728677 PMC6013844

[B6] Challis C. Beck S. G. Berton O. (2014). Optogenetic modulation of descending prefrontocortical inputs to the dorsal raphe bidirectionally bias socioaffective choices after social defeat. Front. Behav. Neurosci. 8:43. 10.3389/fnbeh.2014.00043 24596546 PMC3925846

[B7] Chaudhury D. Walsh J. J. Friedman A. K. Juarez B. Ku S. M. Koo J. W.. (2013). Rapid regulation of depression-related behaviours by control of midbrain dopamine neurons. Nature 493, 532–536. 10.1038/nature11713 23235832 PMC3554860

[B8] Cheng Z. Cui R. Ge T. Yang W. Li B. (2019). Optogenetics: what it has uncovered in potential pathways of depression. Pharmacol. Res. 152:104596. 10.1016/j.phrs.2019.104596 31838082

[B9] Covington H. E. Lobo M. K. Maze I. Vialou V. Hyman J. M. Zaman S.. (2010). Antidepressant effect of optogenetic stimulation of the medial prefrontal cortex. J. Neurosci. 30, 16082–16090. 10.1523/JNEUROSCI.1731-10.2010 21123555 PMC3004756

[B10] Cui W. Aida T. Ito H. Kobayashi K. Wada Y. Kato S.. (2020). Dopaminergic signaling in the nucleus accumbens modulates stress-coping strategies during inescapable stress. J. Neurosci. 40, 7241–7254. 10.1523/JNEUROSCI.0444-20.2020 32847967 PMC7534921

[B11] de Kloet E. R. Molendijk M. L. (2016). Coping with the forced swim stressor: towards understanding an adaptive mechanism. Neural Plast 2016, 1–13. 10.1155/2016/6503162 27034848 PMC4806646

[B12] Duman R. S. Sanacora G. Krystal J. H. (2019). Altered connectivity in depression: GABA and glutamate neurotransmitter deficits and reversal by novel treatments. Neuron 102, 75–90. 10.1016/j.neuron.2019.03.013 30946828 PMC6450409

[B13] Fakhoury M. (2020). Optogenetics: a revolutionary approach for the study of depression. Prog. Neuro Psychopharmacol. Biol. Psychiatry 106:110094. 10.1016/j.pnpbp.2020.110094 32890694

[B14] Friedman A. Homma D. Bloem B. Gibb L. G. Amemori K. Hu D.. (2017). Chronic stress alters striosome-circuit dynamics, leading to aberrant decision-making. Cell 171, 1191–1205. 10.1016/j.cell.2017.10.017 29149606 PMC5734095

[B15] Golden S. A. Covington H. E. Berton O. Russo S. J. (2011). A standardized protocol for repeated social defeat stress in mice. Nat. Protoc. 6, 1183–1191. 10.1038/nprot.2011.361 21799487 PMC3220278

[B16] Hare B. D. Duman R. S. (2020). Prefrontal cortex circuits in depression and anxiety: contribution of discrete neuronal populations and target regions. Mol Psychiatr. 25, 2742–2758. 10.1038/s41380-020-0685-9 32086434 PMC7442605

[B17] Hare B. D. Shinohara R. Liu R. J. Pothula S. DiLeone R. J. Duman R. S. (2019). Optogenetic stimulation of medial prefrontal cortex Drd1 neurons produces rapid and long-lasting antidepressant effects. Nat. Commun. 10:223. 10.1038/s41467-018-08168-9 30644390 PMC6333924

[B18] Hultman R. Mague S. D. Li Q. Katz B. M. Michel N. Lin L.. (2016). Dysregulation of prefrontal cortex-mediated slow-evolving limbic dynamics drives stress-induced emotional pathology. Neuron 91, 439–452. 10.1016/j.neuron.2016.05.038 27346529 PMC4986697

[B19] Iñiguez S. D. Flores-Ramirez F. J. Riggs L. M. Alipio J. B. Garcia-Carachure I. Hernandez M. A.. (2018). Vicarious social defeat stress induces depression-related outcomes in female mice. Biol. Psychiatry 83, 9–17. 10.1016/j.biopsych.2017.07.014 28888327 PMC5730407

[B20] Kataoka N. Shima Y. Nakajima K. Nakamura K. (2020). A central master driver of psychosocial stress responses in the rat. Science 367, 1105–1112. 10.1126/science.aaz4639 32139538

[B21] Krishnan V. Han M.-H. Graham D. L. Berton O. Renthal W. Russo S. J.. (2007). Molecular adaptations underlying susceptibility and resistance to social defeat in brain reward regions. Cell 131, 391–404. 10.1016/j.cell.2007.09.018 17956738

[B22] Kumar S. Black S. J. Hultman R. Szabo S. T. DeMaio K. D. Du J.. (2013). Cortical control of affective networks. J. Neurosci. 33, 1116–1129. 10.1523/JNEUROSCI.0092-12.2013 23325249 PMC3711588

[B23] Newman E. L. Covington H. E. Suh J. Bicakci M. B. Miczek K. A. (2019). Fighting females: neural and behavioral consequences of social defeat stress in female mice. Biol. Psychiatry 86, 657–668. 10.1016/j.biopsych.2019.05.005 31255250 PMC6788975

[B24] Patel D. Kas M. J. Chattarji S. Buwalda B. (2019). Rodent models of social stress and neuronal plasticity: relevance to depressive-like disorders. Behavi. Brain. Res. 36:111900. 10.1016/j.bbr.2019.111900 31022420

[B25] Pryce C. R. Fuchs E. (2017). Chronic psychosocial stressors in adulthood: studies in mice, rats and tree shrews. Neurobiol. Stress 6, 94–103. 10.1016/j.ynstr.2016.10.001 28229112 PMC5314423

[B26] Richardson-Jones J. W. Craige C. P. Nguyen T. H. Kung H. F. Gardier A. M. Dranovsky A.. (2011). Serotonin-1A autoreceptors are necessary and sufficient for the normal formation of circuits underlying innate anxiety. J. Neurosci. 31, 6008–6018. 10.1523/JNEUROSCI.5836-10.2011 21508226 PMC3102496

[B27] Russo S. J. Nestler E. J. (2013). The brain reward circuitry in mood disorders. Nat. Rev. Neurosci. 14, 609–625. 10.1038/nrn3381 23942470 PMC3867253

[B28] Sapolsky R. M. (2016). Psychiatric distress in animals versus animal models of psychiatric distress. Nat. Neurosci. 19, 1387–1389. 10.1038/nn.4397 27786183

[B29] Slattery D. A. Cryan J. F. (2012). Using the rat forced swim test to assess antidepressant-like activity in rodents. Nat. Protoc. 7, 1009–1014. 10.1038/nprot.2012.044 22555240

[B30] Son H. Baek J. H. Go B. S. Jung D. Sontakke S. B. Chung H. J.. (2018). Glutamine has antidepressive effects through increments of glutamate and glutamine levels and glutamatergic activity in the medial prefrontal cortex. Neuropharmacology 143, 143–152. 10.1016/j.neuropharm.2018.09.040 30266598

[B31] Toyoda A. (2017). Social defeat models in animal science: what we have learned from rodent models. Anim. Sci. J. 88, 944–952. 10.1111/asj.12809 28436163 PMC5518448

[B32] Tye K. M. Deisseroth K. (2012). Optogenetic investigation of neural circuits underlying brain disease in animal models. Nat. Rev. Neurosci. 13, 251–266 10.1038/nrn3171 22430017 PMC6682316

[B33] Vialou V. Bagot R. C. Cahill M. E. Ferguson D. Robison A. J. Dietz D. M.. (2014). Prefrontal cortical circuit for depression- and anxiety-related behaviors mediated by cholecystokinin: role of FosB. J. Neurosci. 34, 3878–3887. 10.1523/JNEUROSCI.1787-13.2014 24623766 PMC3951691

[B34] Vos T. Barber R. M. Bell B. Bertozzi-Villa A. Biryukov S. Bolliger I.. (2015). Global, regional, and national incidence, prevalence, and years lived with disability for 301 acute and chronic diseases and injuries in 188 countries, 1990–2013: a systematic analysis for the global burden of disease study 2013. Lancet 386, 743–800. 10.1016/S0140-6736(15)60692-4 26063472 PMC4561509

[B35] Walsh J. J. Han M. H. (2014). The heterogeneity of ventral tegmental area neurons: projection functions in a mood-related context. Neuroscience 282, 101–108. 10.1016/j.neuroscience.2014.06.006 24931766 PMC4339667

[B36] Warden M. R. Selimbeyoglu A. Mirzabekov J. J. Lo M. Thompson K. R. Kim S.-Y.. (2012). A prefrontal cortex–brainstem neuronal projection that controls response to behavioural challenge. Nature 492, 428–432. 10.1038/nature11617 23160494 PMC5929119

